# Identification of key genes and biological pathways in different parts of yak oviduct based on transcriptome analysis

**DOI:** 10.3389/fvets.2022.1016191

**Published:** 2022-11-23

**Authors:** Jian Zhang, Yangyang Pan, Ling Zhao, Tian Zhao, Sijiu Yu, Yan Cui

**Affiliations:** ^1^College of Veterinary Medicine, Gansu Agricultural University, Lanzhou, China; ^2^Technology and Research Center of Gansu Province for Embryonic Engineering of Bovine and Sheep & Goat, Lanzhou, China

**Keywords:** yak, oviduct, transcriptome, hub genes, key pathways

## Abstract

The oviduct consists of three parts: the infundibulum (In), ampulla (Am), and isthmus (Is). These have the same histological structure, but different physiological functions. In this study, transcriptomics was used to analyze mRNA in these three parts of yak oviduct. The results showed that there were 325 up-regulated genes and 282 down-regulated genes in the infundibulum and ampulla. Moreover, there were 234 up-regulated genes and 776 down-regulated genes in the isthmus and ampulla, as well as 873 up-regulated genes and 297 down-regulated genes in the infundibulum and isthmus. The expression of C3 in the infundibulum was significantly higher than that in the ampulla and isthmus. The expression of FAU in the isthmus was significantly lower than that in the ampulla and infundibulum, and the expression of EEF1A1 in the ampulla was significantly higher than that in the ampulla and infundibulum. When the infundibulum was compared with the ampulla and isthmus, it was found that the up-regulated genes were enriched in the lysosome, phagosome, staphylococcus aureus infection, and leishmaniasis pathway. When the isthmus was compared with the ampulla and infundibulum, the up-regulated genes were present in the apoptosis pathway, oxidative phosphorylation, and viral myocarditis pathway. When the isthmus was compared with the infundibulum and ampulla, the down-regulated pathways were protein processing in the endoplasmic reticulum and the endocytosis. The Epstein–Barr virus infection pathway was up-regulated according to a comparison of the isthmus and infundibulum and was down-regulated based on a comparison of the isthmus and ampulla. Transcriptional misregulation in the Middle East pathway was up-regulated based on a comparison of the isthmus and ampulla and was down-regulated based on a comparison of the isthmus and infundibulum. ERBB2, JUP, CTNND1, and KRT7 were defined as the hub genes of the yak oviduct. The results of this study provide sufficient omics data for yak fertilization, which is also of great significance to altitude medicine.

## Introduction

The oviduct is a pair of thin, curved muscular tubes comprising smooth muscle. It is the primary location where oocytes and sperm meet. The oviduct is composed of three parts: the infundibulum, ampulla, and isthmus. These have similar histological structures, but different physiological functions *in vivo*. The expression of oviduct-specific mRNAs varies in different regions (1); VEGF, FGF1, FGF2, and their receptors are expressed in all three parts of the equine oviduct (2). Hormones also have important effects on oviduct movement, particularly in the follicular phase; OTR and oxytocin exhibit significant stimulatory effects on cow oviduct movement (3). There have been many studies on the oviduct; however, research on different parts of the oviduct in yaks remains unexplored. As such, our study is the first to analyze mRNA in different parts of the yak oviduct using transcriptomic analysis. This study provides an opportunity to discuss the physiological functions of different parts of the oviduct from the perspective of mRNA.

Oviduct epithelial cells are composed of ciliated and secretory cells; the ratio of ciliated to secretory cells is different in the three distinct parts of the oviduct (4). Epithelial cells can change periodically upon regulation by estradiol and progesterone (5, 6); estradiol can regulate the expressions of SBD-1 (7), S100A8 (8), prostaglandin E2 (PGE2), prostaglandin F2α (PGF2α) (9), and the melatonin receptors MT1 and MT2 (10) in oviduct epithelial cells. The innermost layer of the oviduct is made up of epithelial cells, and the middle layer is muscle. The thickness of the muscular layer is different in the three parts of the oviduct. The muscular layer is thickest in the isthmus, particularly during estrus, and the contraction of the muscular layer makes the oviduct lumen significantly smaller (11). The muscular layer plays an important role in oviduct peristalsis; it can extend peristalsis from the infundibulum to the isthmus and *vice versa*. Most importantly, the oviduct isthmus is curved and folded which could provide a location for sperm storage, and its epithelial cells contribute to sperm motility (12, 13). The apex of the isthmus cilia and the sperm flagellum initiate special activity reactions to regulate sperm motility (14).

At present, there are many studies on the oviduct, sperm, oocytes, and zygotes, including the regulation of bovine oviduct epithelial cells by E_2_ (15), and the regulation of porcine oviduct by hCG and eCG (16). However, studies on the three parts of the oviduct itself are lacking. In this study, transcriptomics was used to explore the hub genes and key pathways, which regulate different physiological functions of the oviduct, from the perspective of mRNA. This study presents sufficient omics data for the analysis of mRNA in yak oviducts, providing new views on the study of the fertilization mechanism in plateau animals.

## Materials and methods

### Animal materials

All experiments were approved by the Animal Ethics Committee of Gansu Agricultural University. Clinically healthy adult yaks were selected from the grasslands of Xining, China, at an altitude of 3,800 m; the yaks were euthanized by administering pentobarbital sodium (200 mg/kg) *via* intravenous injection. One oviduct was collected from each yak and two adult female yaks were selected for single-cell experiments. The oviduct tissue was washed with sterile saline, placed in a tissue-protection fluid, and transported to the laboratory for subsequent RNA sequencing. The oviduct tissue was cut into 1 mm^3^ cubes and digested with collagenase (GIBCO) and trypsin (GIBCO) for 25 min (containing 2 mmol EDTA). Digestion was then stopped and the oviduct tissue was filtered. Cells were washed twice with 10% BSA to obtain a single-cell suspension and RNA sequencing was performed on a Chromium.

### Identification of differentially expressed genes

We integrated RNA sequencing data and compiled a gene expression matrix for each of the three parts of the oviduct. The mRNA of the infundibulum (In), ampulla (Am), and isthmus (Is) were compared to explore the potential differentially expressed genes (DEGs). Furthermore, the up-regulated DEGs and down-regulated DEGs were distinguished. To demonstrate the good repeatability and usability of the data, the DEGs were visualized as volcano plots and heatmaps by using the R package, which were drawn for the infundibulum and ampulla group, the isthmus and ampulla group, and the infundibulum and isthmus group. |Log_2_FC| > 2 and an FDR significance score of <0.05 were used for the DEGs and subsequent analysis.

### GO-KEGG enrichment analysis

In order to show the physiological pathways of up-regulated and down-regulated DEGs, we enriched the biological functions. GO and KEGG are databases mainly for gene function and enrichment, respectively. GO analysis covers molecular function (MF), biological process (BP), and cellular component (CC). KEGG focuses on the pathway. DEGs were mapped to the KEGG database (Kyoto Encyclopedia of Genes and Genomes) for Bos mutus (wild yak, database ID: T02919 at https://www.genome.jp) (17). The enrichment of DEGs was performed using GO-KEGG analyses with a value of *P* < 0.05.

### PPI network and hub genes analysis

To illustrate the protein-protein interaction, we used a string database (https://string-db.org/) to retrieve the potential interaction of the encoded protein and built the protein-protein interaction network. In the regulatory network, nodes represent proteins, and the node labels were the names of these proteins. The pattern in the node represents the three-dimensional structure of the protein, which is unknown if empty. Protein-protein interaction networks helped us to understand the interactions between proteins, explore the hub genes of regulatory networks, and identify the physiological function. Using the cytoHubba plugin in Cytoscape 3.7.1, we screened the top 10 hub genes and ranked them based on the scores.

## Results

### Identification of DEGs

**Figure 1:**
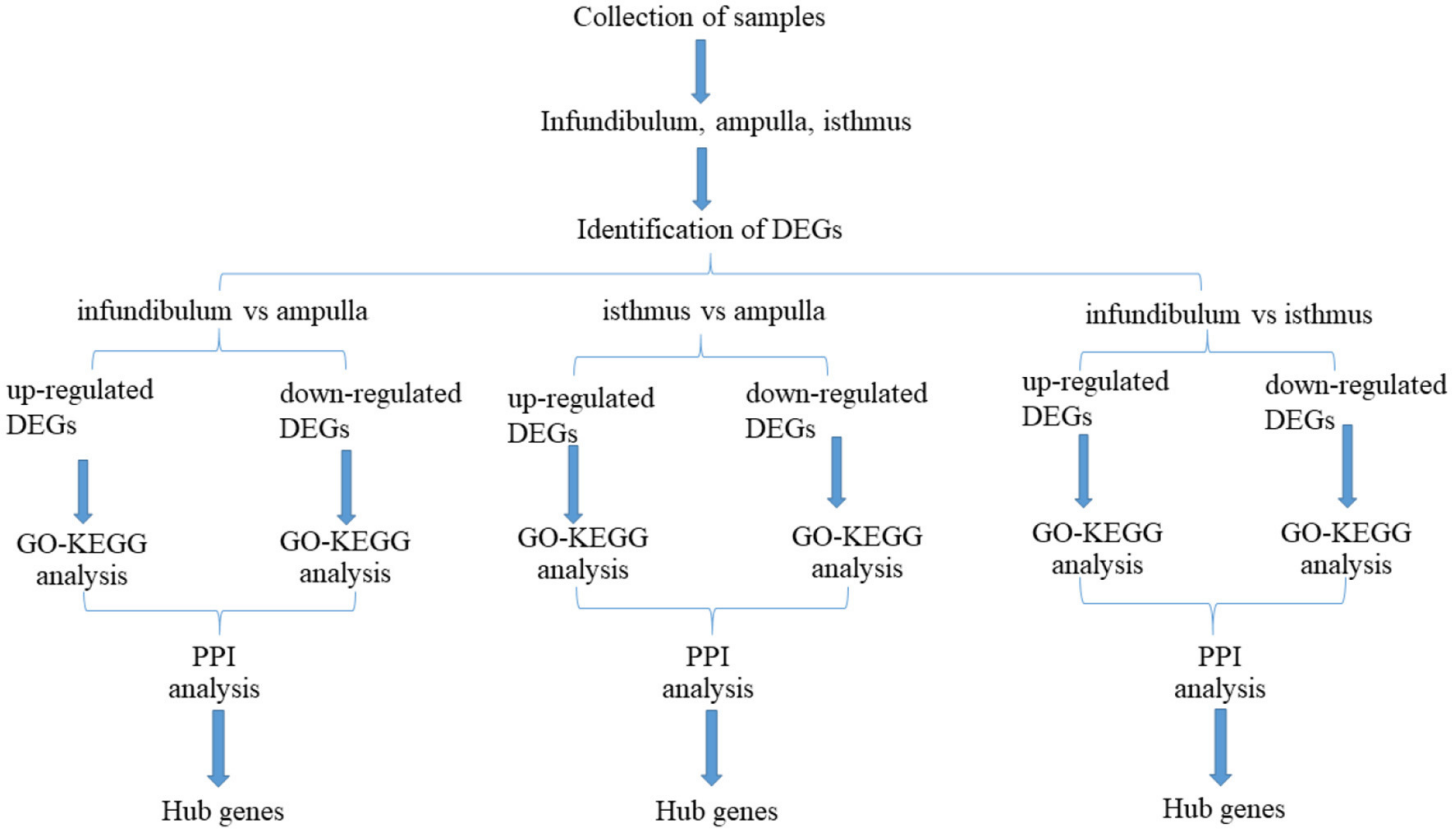
Flow chart for this study.

**Figure 2:**
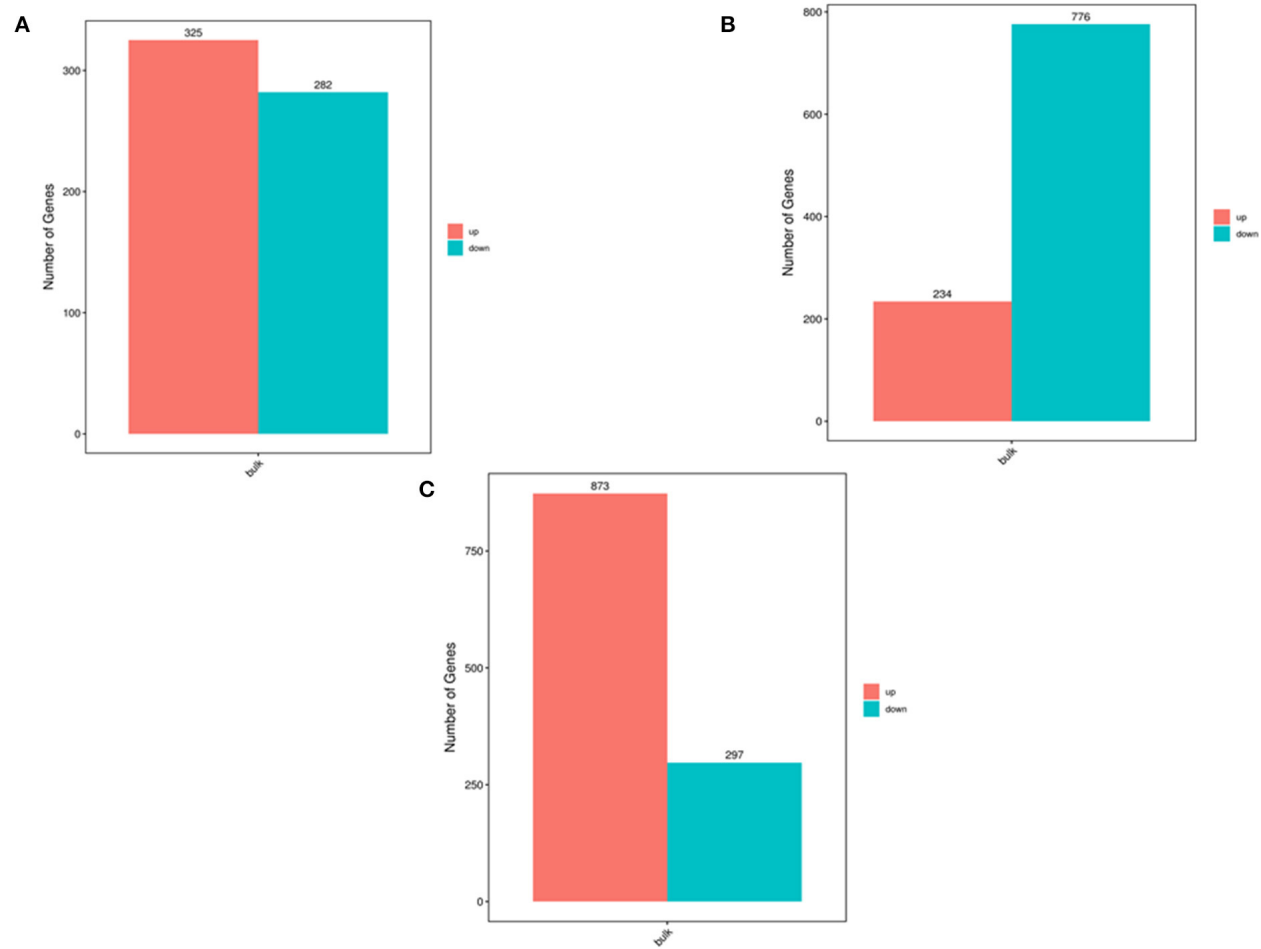
The number of DEGs determined by transcriptome analysis. **(A)** Number of DEGs between the infundibulum and ampulla. **(B)** Number of DEGs between the isthmus and ampulla. **(C)** Number of DEGs between the infundibulum and isthmus.

Figure 1 shows a flow chart of the entire experimental process. The three parts of the oviduct were compared. It was found that there were 325 up-regulated genes and 282 down-regulated genes based on a comparison of the infundibulum and ampulla (Figure 2A). There were 234 up-regulated genes and 776 down-regulated genes according to a comparison of the isthmus and ampulla (Figure 2B), and 873 up-regulated genes and 297 down-regulated genes based on a comparison of the infundibulum and isthmus (Figure 2C).

**Figure 3:**
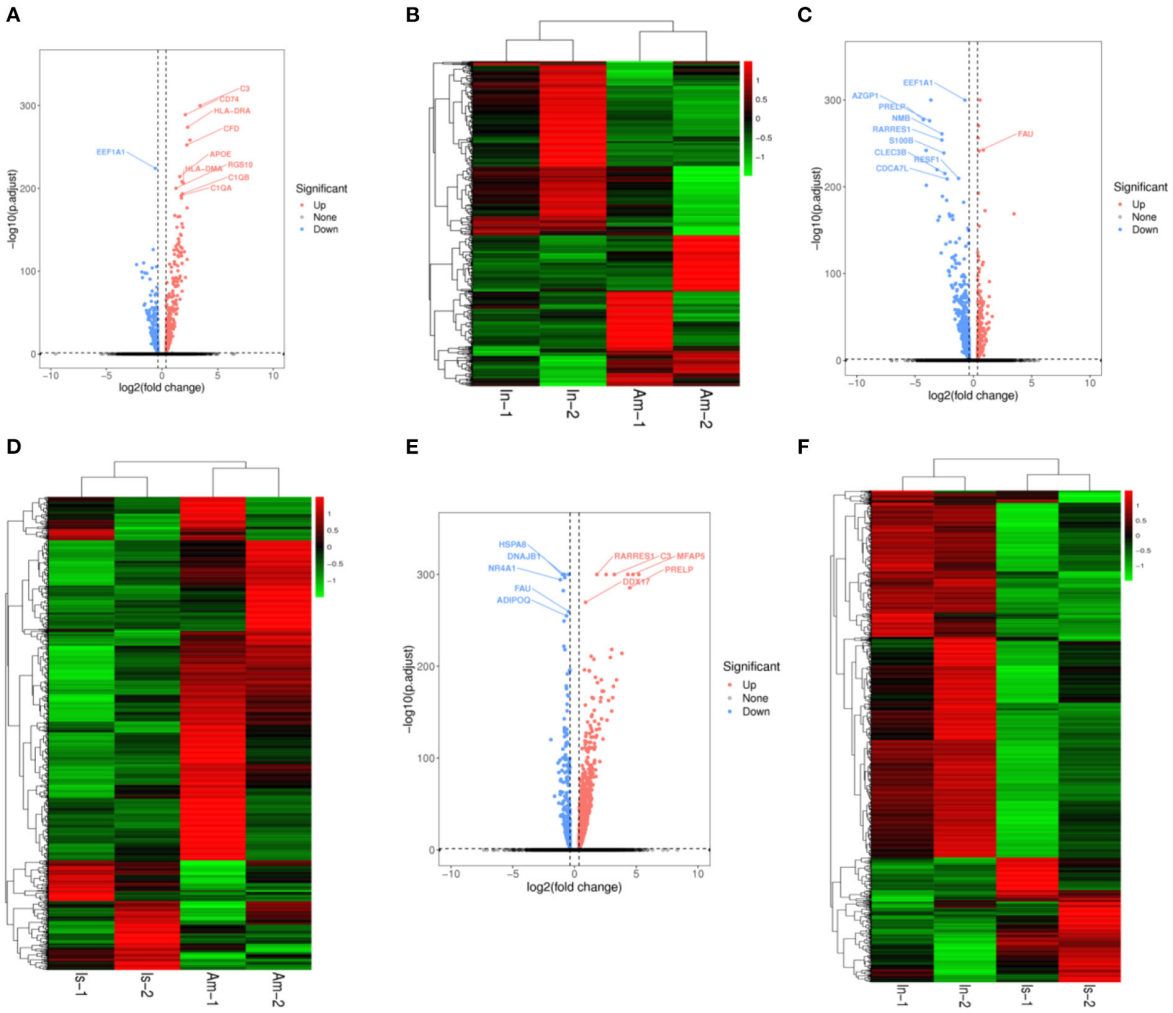
Volcano plots and heatmaps in the yak oviduct. **(A)** Volcano plot of the infundibulum and ampulla. **(B)** Heatmap of the infundibulum and ampulla. **(C)** Volcano plot of the isthmus and ampulla. **(D)** Heatmap of the isthmus and ampulla. **(E)** Volcano plot of the infundibulum and isthmus. **(F)** Heatmap of the infundibulum and isthmus.

We compared the infundibulum with the ampulla and constructed volcano plots (Figure 3A) and heat maps (Figure 3B). After comparison, it was found that C3, CD74, HLA-DRA, and CFD were the up-regulated genes exhibiting significant differences, and EEF1A1 was the down-regulated gene displaying significant differences. The isthmus was compared with the ampulla, and the volcano plot (Figure 3C) and heat map (Figure 3D) were drawn. FAU was the up-regulated gene presenting significant differences, while EEF1A1, AZGP1, and PRELP were the down-regulated genes showing significant differences. The infundibulum was compared with the isthmus, the results are shown in the volcano plot (Figure 3E) and heatmap (Figure 3F). RARRES1, C3, MFAP5 HSPA8, DNAJB1, and NR4A1 were the up-regulated genes displaying significant differences. FAU was a significantly different down-regulated gene.

Based on the above results, C3 was an up-regulated gene exhibiting significant differences when the infundibulum was compared with the ampulla and isthmus. FAU was a down-regulated gene presenting significant differences when the isthmus was compared with the ampulla and infundibulum.

### Enrichment of DEGs

**Figure 4:**
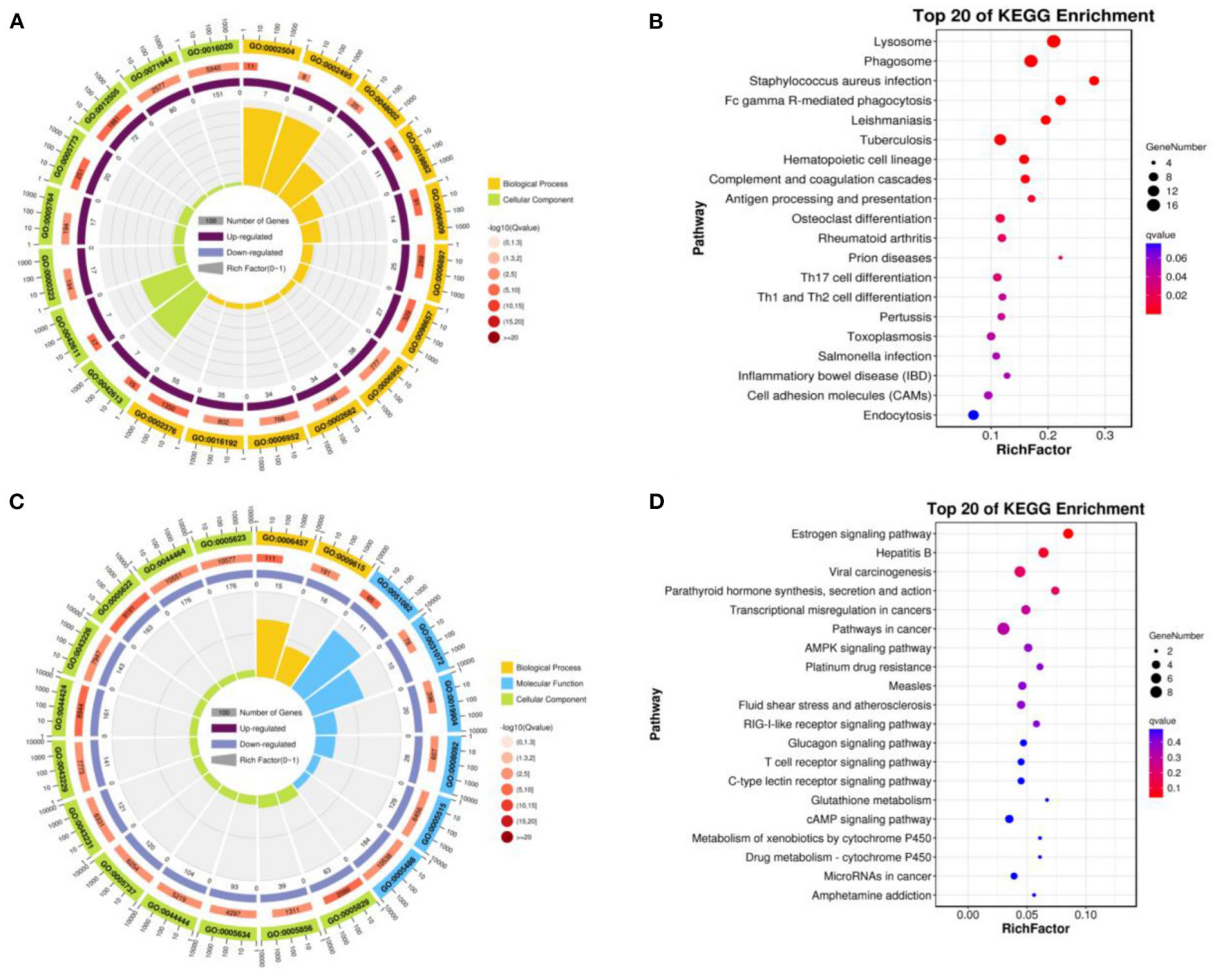
Function of enrichment in the infundibulum and ampulla. **(A)** GO analysis of the up-regulated DEGs. **(B)** Pathways analysis of the up-regulated DEGs. **(C)** GO analysis of the down-regulated DEGs. **(D)** Pathways analysis of the down-regulated DEGs.

Up-regulated DEGs were obtained by functional enrichment analysis after comparison of the infundibulum and ampulla. The biological processes included the cellular process, biological regulation, metabolic process, regulation of the biological process, response to stimulus, localization, cellular component organization or biogenesis, multicellular organismal process, positive regulation of the biological process, signaling, developmental process, negative regulation of the biological process, and the immune system process. The cellular components were mainly rich in cells, cell component, organelle, membrane, membrane component, organelle component, protein-containing complex, and membrane-enclosed lumen. The molecular functions were binding, catalytic activity, and the molecular function regulator (Figure 4A). These up-regulated differential genes reflected the lysosome pathway, phagosome pathway, staphylococcus aureus infection pathway, fc gamma R-mediated phagocytosis pathway, leishmaniasis pathway, tuberculosis pathway, and the hematopoietic cell lineage pathway (Figure 4B).

Down-regulated DEGs were obtained after comparison of the infundibulum and ampulla, with biological processes covering the cellular process, biological regulation, regulation of the biological process, metabolic process, response to stimulus, positive regulation of the biological process, cellular component organization or biogenesis, negative regulation of the biological process, signaling, localization, multicellular organismal process, and the developmental process. The cellular component covered the cell, cell component, organelle, organelle component, membrane, membrane-enclosed lumen, protein-containing complex, and the membrane component. The molecular functions were binding, catalytic activity, transcription regulator activity, and the molecular function regulator (Figure 4C). These down-regulated DEGs included the estrogen signaling pathway, hepatitis B, viral carcinogenesis, parathyroid hormone synthesis, and the secretion and action pathway (Figure 4D).

**Figure 5:**
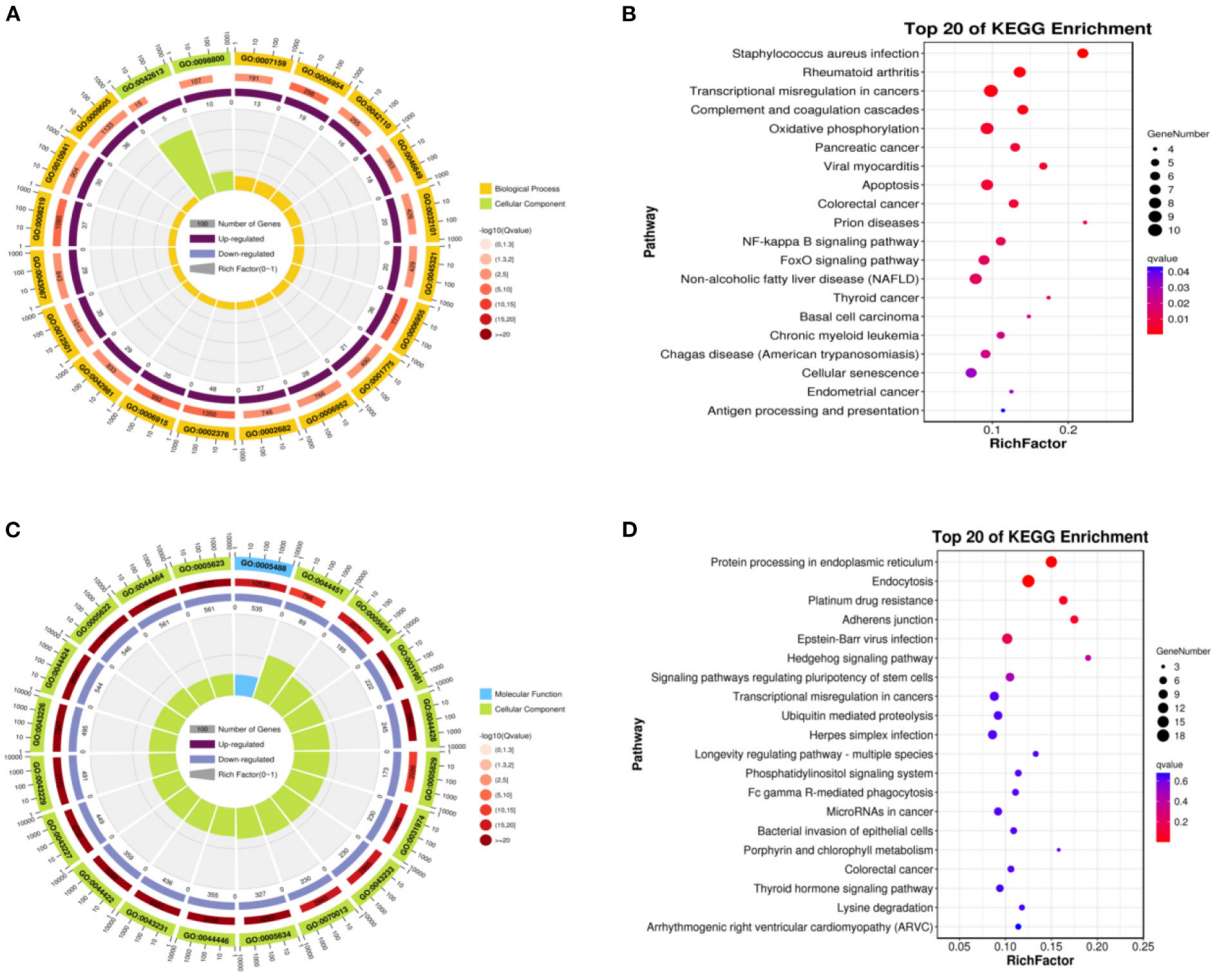
Function of enrichment in the isthmus and ampulla. **(A)** GO functional enrichment of the up-regulated DEGs. **(B)** Pathways enrichment of the up-regulated DEGs. **(C)** GO functional enrichment of the down-regulated DEGs. **(D)** Pathways enrichment of the down-regulated DEGs.

We compared the isthmus with the ampulla, the up-regulated DEGs were obtained. GO-KEGG analysis was then performed on up-regulated DEGs. The biological processes covered the cellular process, biological regulation, metabolic process, regulation of the biological process, response to stimulus, positive regulation of the biological process, localization, cellular component organization or biogenesis, negative regulation of the biological process, signaling, multicellular organismal process, immune system process, and the developmental process. The cellular components were the cell, cell component, organelle, membrane, protein-containing complex, organelle component, membrane component, and membrane-enclosed lumen. The molecular function included binding, catalytic activity, and the molecular function regulator (Figure 5A). These up-regulated DEGs included staphylococcus aureus infection, rheumatoid arthritis, transcriptional misregulation in cancers, complement and coagulation cascades, oxidative phosphorylation, pancreatic cancer, viral myocarditis, and the apoptosis pathway (Figure 5B).

We obtained down-regulated DEGs after comparison of the isthmus and ampulla. The biological processes included the cellular process, metabolic process, biological regulation, regulation of the biological process, response to stimulus, cellular component organization or biogenesis, negative regulation of the biological process, positive regulation of the biological process, localization, multicellular organismal process, developmental process, and signaling. The cellular components included the cell, cell component, organelle, organelle component, membrane-enclosed lumen, protein-containing complex, membrane, and the membrane component. The molecular functions were mainly binding, catalytic activity, transcription regulator activity, and the molecular function regulator (Figure 5C). These down-regulated DEGs included protein processing in the endoplasmic reticulum, endocytosis, platinum drug resistance, adherens junction, Epstein–Barr virus infection, hedgehog signaling pathway, and the signaling pathways regulating the pluripotency of stem cells (Figure 5D).

**Figure 6:**
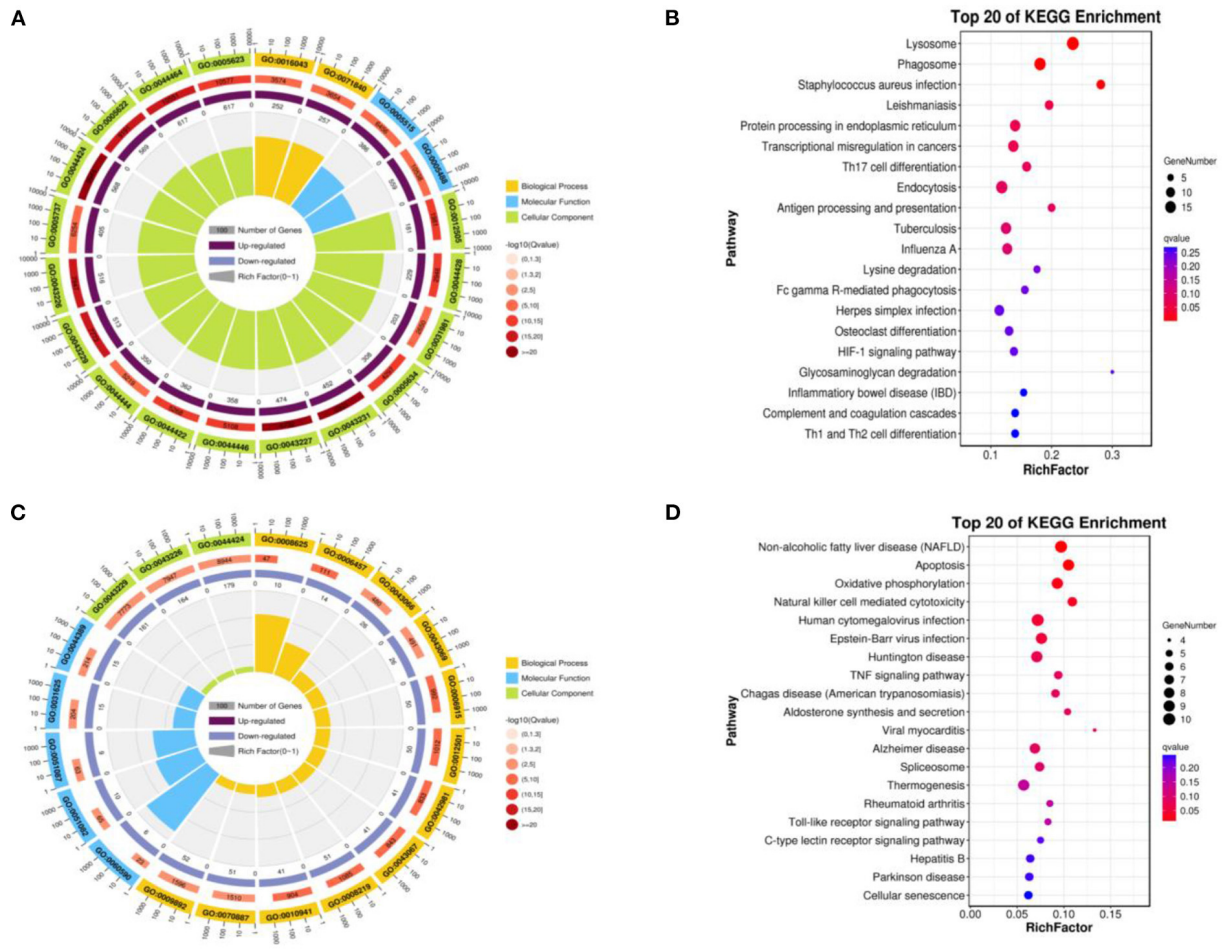
Function of enrichment in the infundibulum and isthmus. **(A)** GO enrichment of the up-regulated DEGs. **(B)** Biological pathways of the up-regulated DEGs. **(C)** GO enrichment of the down-regulated DEGs. **(D)** Biological pathways enrichment of the down-regulated DEGs.

We compared the infundibulum and isthmus, and the up-regulated DEGs were obtained. The biological processes covered the cellular process, metabolic process, biological regulation, regulation of the biological process, response to stimulus, cellular component organization or biogenesis, localization, positive regulation of the biological process, multicellular organismal process, negative regulation of the biological process, developmental process, and signaling. The cellular components were the cell, cell component, organelle, organelle component, membrane, protein-containing complex, membrane-enclosed lumen, and the membrane component. The molecular function was mainly enriched in binding, catalytic activity, molecular function regulator, and transcription regulator activity (Figure 6A). These up-regulated DEGs included the lysosome, phagosome, staphylococcus aureus infection, leishmaniasis, protein processing in endoplasmic reticulum, transcriptional misregulation in cancers, th17 cell differentiation, endocytosis, antigen processing and presentation, tuberculosis, and the influenza A pathway (Figure 6B).

The down-regulated DEGs were obtained by comparing the infundibulum with the isthmus, and subsequently, GO-KEGG analysis was performed on these genes. The biological processes were closely related to the cellular process, metabolic process, biological regulation, regulation of the biological process, response to stimulus, positive regulation of the biological process, negative regulation of the biological process, cellular component organization or biogenesis, signaling, localization, multicellular organismal process, developmental process, immune system process, multi-organism process, and cell proliferation. The cellular components covered the cell, cell component, organelle, organelle component, protein-containing complex, membrane, membrane-enclosed lumen, and the membrane component. The molecular functions were mainly enriched in binding, catalytic activity, molecular function regulator, transcription regulator activity, structural molecule activity, transporter activity, and the molecular transducer activity (Figure 6C). These down-regulated DEGs were closely related to non-alcoholic fatty liver disease (NAFLD), apoptosis, oxidative phosphorylation, natural killer cell-mediated cytotoxicity, human cytomegalovirus infection, Epstein–Barr virus infection, Huntington's disease, TNF signaling pathway, Chagas disease (American trypanosomiasis), aldosterone synthesis and secretion, and viral myocarditis (Figure 6D).

### Analysis of PPI regulation

**Figure 7:**
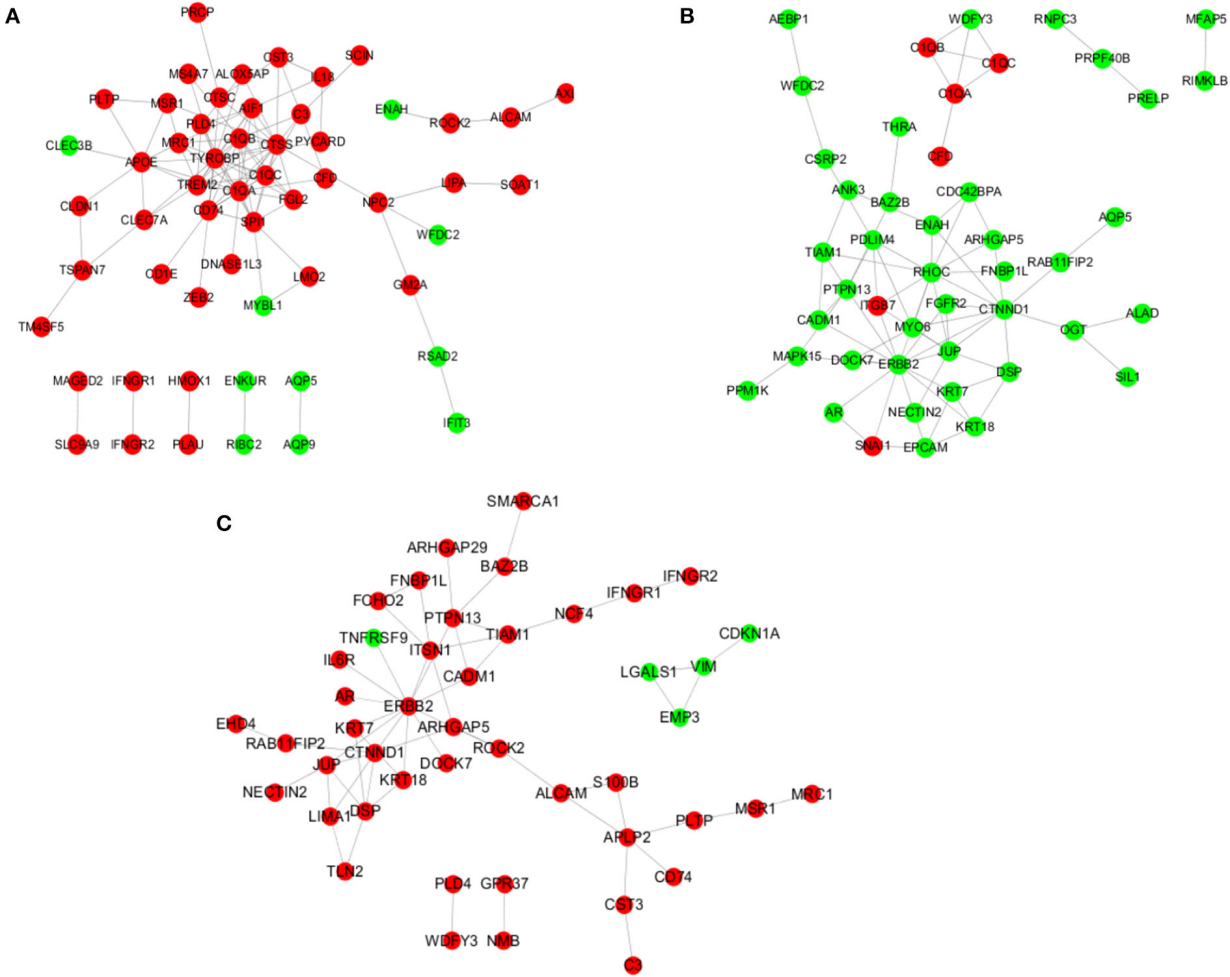
Analysis of the regulatory network. **(A)** Regulatory network in the infundibulum and ampulla group. **(B)** Regulatory network in the isthmus and ampulla group. **(C)** Regulatory network in the infundibulum and isthmus group.

When the infundibulum was compared with the ampulla (Figure 7A), C3, CD74, RGS10, C1QB, C1QA, C1QC, CST3, AIF1, TIMD4, GM2A, TYROBP, XDH, and so on, were the up-regulated DEGs, while CLEC3B, ENAH, WFDC2, MYBL1, ENKUR, RIBC2, AQP5, AQP9, RSAD2, and IFIT3 were the down-regulated DEGs. AIF1 regulated MRC1, CTSS, TREM2, IL18, PLD4, C1QB, TYROBP, and C1QA. APOE regulated MSR1, MRC1, CLEC3B, TREM2, PLTP, CLDN1, CLEC7A, and C1QA. C1QA regulated C3, MRC1, CTSS, FGL2, TREM2, SPI1, CD74, C1QB, CFD, TYROBP, and C1QC. CD74 regulated CD74, ZEB2, CTSS, SPI1, TYROBP, and CTSC. C3 regulated CST3, SCIN, and CFD.

When the isthmus was compared with the ampulla (Figure 7B), AEBP1, WFDC2, CSRP2, ANK3, WDFY3, RNPC3, MFAP5, PRPF40B, RIMKL, and so on, were the down-regulated DEGs. C1QB, C1QC, C1QA, CFD, ITGB7, and SNAI1 were the up-regulated DEGs. ARHGAP5 regulated RHOC, CTNND1, and CDC42BPA. C1QA regulated WDFY3, C1QB, CFD, and C1QC. CTNND1 regulated RHOC, FGFR2, RAB11FIP2, MYO6, OGT, DSP, ENAH, JUP, and ERBB2. ERBB2 regulated RHOC, MAPK15, FGFR2, ITGB7, KRT18, PTPN13, SNAI1, KRT7, and JUP. ITGB7 regulated RHOC, ITGB7, PDLIM4, ITGB7, and JUP.

When the infundibulum was compared with the isthmus (Figure 7C), SMARCA1, ARHGAP29, BAZ2B, FNBP1L, IFNGR2, IFNGR1, FCHO2, PTPN13, TIAM1, and so on, were the up-regulated DEGs. TNFRSF9, LGALS1, EMP3, CDKN1A, and VIM were the down-regulated DEGs. APLP2 regulated CD74, S100B, CST3, and PLTP. ALCAM regulated APLP2, ROCK2, and S100B. CTNND1 regulated RAB11FIP2, DSP, LIMA1, JUP, and ERBB2. ERBB2 regulated ROCK2, TNFRSF9, ITSN1, KRT18, PTPN13, KRT7, IL6R, and JUP. DSP regulated TLN2, KRT18, and KRT7. JUP regulated LIMA1 and NECTIN2. IFNGR1 regulated NCF4 and IFNGR2.

### Identification of hub genes

**Figure 8:**
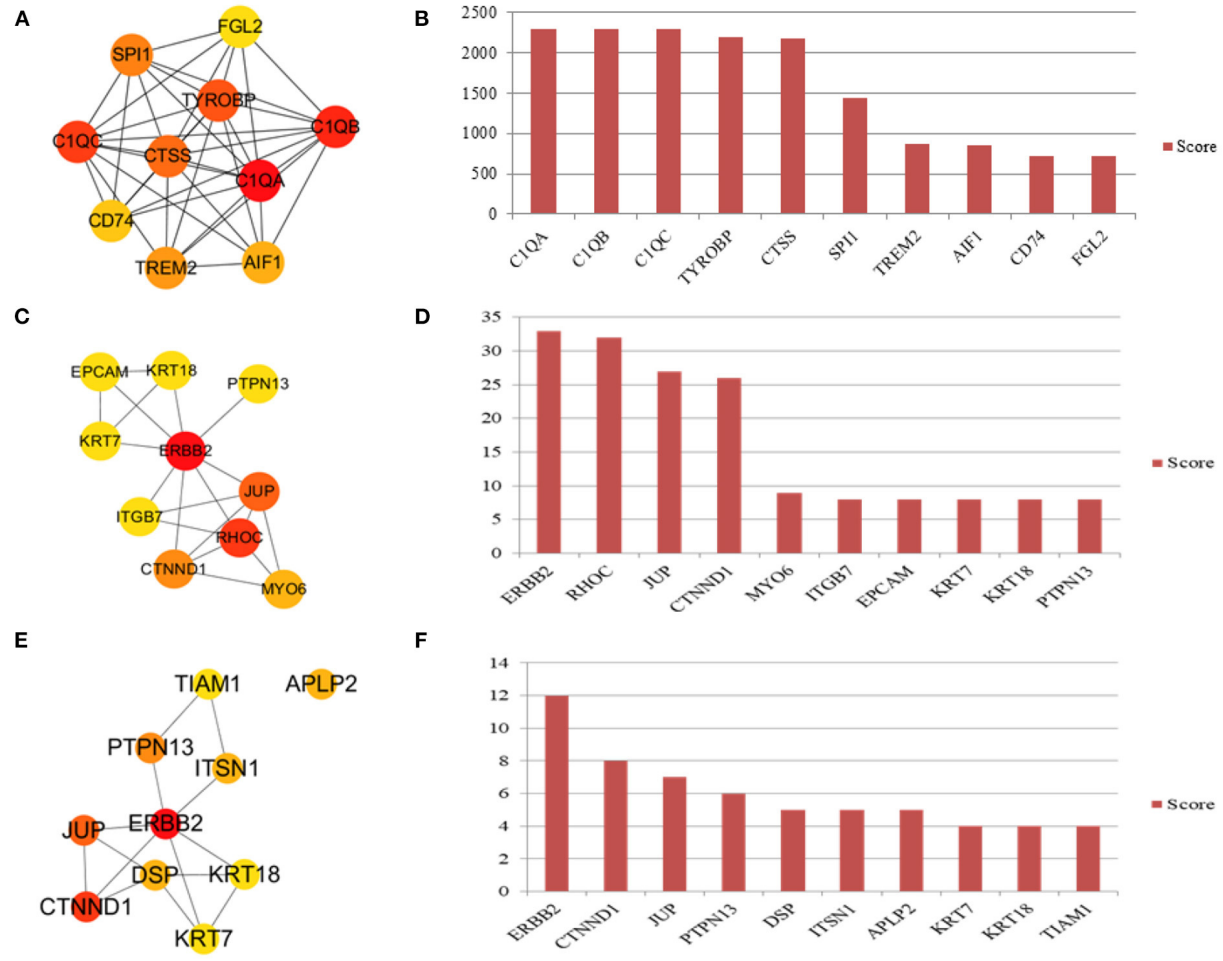
Hub genes in the yak oviduct. **(A)** Hub genes of the infundibulum and ampulla. **(B)** The rank of hub genes between the infundibulum and ampulla. **(C)** Hub genes of the isthmus and ampulla. **(D)** The rank of hub genes between the isthmus and ampulla. **(E)** Hub genes of the infundibulum and isthmus. **(F)** The rank of hub genes between the infundibulum and isthmus.

The infundibulum and ampulla were compared to identify the top 10 hub genes in the regulatory network (Figure 8A), and the rank was as follows: C1QA, C1QB, C1QC, TYROBP, CTSS, SPI1, TREM2, AIF1, CD74, and FGL2 (Figure 8B). The isthmus and ampulla were compared to determine the hub genes in the regulatory network (Figure 8C), and the rank was as follows: ERBB2, RHOC, JUP, CTNND1, MYO6, ITGB7, EPCAM, KRT7, KRT18, and PTPN13 (Figure 8D). The hub genes were screened in the regulatory network by comparing the infundibulum and isthmus (Figure 8E), and the rank was as follows: ERBB2, CTNND1, JUP, PTPN13, DSP, ITSN1, APLP2, KRT7, KRT18, and TIAM1 (Figure 8F).

## Discussion

The oviduct is an important site for fertilization in yaks and previous studies focused on its morphology (18), POxia-inducible factor-1 alpha (19), ERK1/2 (20), estrogen receptor genes (21), follicle-stimulating hormone receptor (FSHR) gene (22), DNA damage induced transcript 3 (23), and apoptosis-related genes in the yak oviduct (24). There are currently no transcriptomic studies on the different parts of the yak oviduct, and there is a lack of research on the fertilization mechanism from the perspective of mRNA. Therefore, transcriptomic studies on the infundibulum, ampulla, and isthmus were completed to explore the hub genes and pathways in yak oviducts.

The infundibulum, ampulla, and isthmus have similar histological structures, but their physiological functions are quite different. C3 was an up-regulated gene that was more significantly expressed in the infundibulum than in the ampulla and isthmus. C3 was also highly expressed before sperm or embryo implantation (25), which may be related to the regulation of E_2_ before embryo implantation (26). In human oviduct epithelial cell cultures, the oviduct epithelial cells produced C3, and C3 immunoreactivity was present (27), and in this study, the most obvious high expression was that of C3 in the infundibulum region. It was speculated that C3 immunoreactivity in the infundibulum component of the oviduct may be the most significant. The NR4A1 gene could be a molecular marker linked to increased litter sizes in pigs (28). In this study, NR4A1 was significantly down-regulated in the infundibulum region compared with the ampulla region. In the study of yak productivity, whether the NR4A1 gene can be used as a marker gene for productivity improvement requires further investigation. HSPA8 was an active component in the oviduct of ewes (29) and could interact with gametes (30). HSPA8 regulated polyspermy modulation and embryonic development (31), and after spermatozoa-oviduct co-culture, the expressions of ADM, HSPA8, and PGES decreased significantly in oviductal epithelial cells (32). These results suggest that HSPA8 was useful in aiding the survival of sperm in the oviduct (33); the addition of HSPA8 could prolong the *in vitro* storage of ram sperm (34). In this study, HSPA8 was significantly higher in the isthmus than in the infundibulum, suggesting that it was related to sperm storage and movement in the isthmus, consistent with previous findings.

Mitochondrial oxidative phosphorylation plays an important role in the migration of sperm from the uterus to the oviduct (35). In this study, the isthmus exhibited higher expressions than the other two sites; the isthmus is the location where sperm transport and storage occur. It was inferred that the isthmus of yaks is also important for sperm transport, similar to other animals. Female parent-bound embryo development occurs by inducing apoptosis of oviductal cells (36), and a growth hormone regulates the apoptosis of oviduct cells and the expression of certain oviduct-specific proteins (37). Moreover, ceramide induces apoptosis in oviduct-derived primary cells *via* a caspase-3– and bcl-2–dependent pathway (38). Apoptosis is particularly important in the study of the yak oviduct; therefore, the specific mechanism of apoptosis requires further study. In the examination of hub genes, ERBB2, JUP, CTNND1, and KRT7 were regarded as hub regulatory genes. MUC4/SMC and ErbB2 were associated with different tissues of the female reproductive tract. Interestingly, phosphorylated ErbB2 was mainly present on the apical surface of the oviduct (39). KRT7 played an important role in the differentiation of oviduct cells (40), and the specific differentiation mechanism requires further study.

In summary, this study is the first time to analyze hub genes and key pathways in three parts of the oviduct using transcriptomics methods. We also analyzed the regulatory network in the oviduct from the perspective of mRNA. This work provides a new view on the study of plateau animals, and a new method for the study of fertilization mechanisms.

## Data availability statement

The authors acknowledge that the transcriptome data stored in a database of GSA (https://ngdc.cncb.ac.cn/gsa/), the data under accession CRA007411 are publicly available.

## Ethics statement

The animal study was reviewed and approved by the Animal Ethics Committee of Gansu Agricultural University. Written informed consent was obtained from the owners for the participation of their animals in this study.

## Author contributions

JZ and YC conceived the project. JZ and YP wrote the manuscript with the help of all authors, and SY revised the manuscript. LZ, SY, and TZ performed transcriptome sequencing. All authors contributed to the writing and approval of the submitted version.

## Funding

This research was supported by the National Natural Science Foundation of China (Grant Nos. 31972634 and 32273091) and the Gansu Provincial Education Department Industry Support Guidance Project (2019C-03).

## Conflict of interest

The authors declare that the research was conducted in the absence of any commercial or financial relationships that could be construed as a potential conflict of interest.

## Publisher's note

All claims expressed in this article are solely those of the authors and do not necessarily represent those of their affiliated organizations, or those of the publisher, the editors and the reviewers. Any product that may be evaluated in this article, or claim that may be made by its manufacturer, is not guaranteed or endorsed by the publisher.
